# The effectiveness of malaria camps as part of the malaria control program in Odisha, India

**DOI:** 10.1038/s41598-023-46220-x

**Published:** 2023-12-28

**Authors:** Danielle C. Ompad, Timir K. Padhan, Anne Kessler, Yesim Tozan, Abbey M. Jones, Anna Maria van Eijk, Steven A. Sullivan, Mohammed A. Haque, Madan Mohan Pradhan, Sanjib Mohanty, Jane M. Carlton, Praveen K. Sahu

**Affiliations:** 1 School of Global Public Health, New York University, New York, NY 10003 USA; 2 Department of Molecular Biology and Infectious Diseases, Community Welfare Society Hospital, Rourkela, Odisha 769042 India; 3 Center for Genomics and Systems Biology, Department of Biology, New York University, New York, NY 10003 USA; 4 Liverpool School of Tropical Medicine, Liverpool, United Kingdom; 5 Department of Health and Family Welfare, State Vector Borne Disease Control Programme, Bhubaneswar, Odisha 751001 India; 6 Johns Hopkins Malaria Research Institute, Johns Hopkins Bloomberg School of Global Public Health, Baltimore, MD 21205 USA

**Keywords:** Malaria, Epidemiology

## Abstract

Durgama Anchalare Malaria Nirakaran (DAMaN) is a multi-component malaria intervention for hard-to-reach villages in Odisha, India. The main component, malaria camps (MCs), consists of mass screening, treatment, education, and intensified vector control. We evaluated MC effectiveness using a quasi-experimental cluster-assigned stepped-wedge study with a pretest–posttest control group in 15 villages: six immediate (Arm A), six delayed (Arm B), and three previous interventions (Arm C). The primary outcome was PCR + *Plasmodium* infection prevalence. The time (i.e., baseline vs. follow-up 3) x study arm interaction term shows that there were statistically significant lower odds of PCR + *Plasmodium* infection in Arm A (AOR = 0.36, 95% CI = 0.17, 0.74) but not Arm C as compared to Arm B at the third follow-up. The cost per person ranged between US$3–8, the cost per tested US$4–9, and the cost per treated US$82–1,614, per camp round. These results suggest that the DAMaN intervention is a promising and financially feasible approach for malaria control.

## Introduction

India has made noteworthy progress towards malaria elimination, with cases decreasing from 20 million in 2000 to approximately 4.1 million cases in 2020^1^. Despite this decline over the past two decades, it remains an important public health problem. India was one of eleven countries accounting for 70% of the global burden of malaria in 2020^1^, and it continues to account for 79% of all malaria cases and 83% of all malaria deaths in the South-East Asia region^2^. Within India, the state of Odisha has the highest burden of malaria, accounting for 22.4% of all cases in the country in 2020, of which 91.4% were *Plasmodium falciparum* infections^3^. The malaria burden in Odisha has been persistently high in the remote, forested areas of the state. In response to this, the Government of Odisha implemented the Durgama Anchalare Malaria Nirakaran (DAMaN; ‘malaria control in inaccessible areas’) program in 2017. DAMaN was designed to supplement existing and routine malaria control programs that serve approximately 5000 inaccessible villages and hamlets in the rural parts of the state^4^.

A key activity of the DAMaN program is the implementation of ‘malaria camps’ (MCs). In the MC model, teams of health workers visit villages and hamlets to provide the intervention which includes seven key activities in each cycle: (1) one round of mass screen and treat (MSAT) before the monsoon season conducted with point-of-care rapid diagnostic tests (RDTs), (2) one or two rounds of fever screen and treat (FSAT) during or after the monsoon season, (3) IRS (indoor residual spraying), (4) other vector control methods, (5) LLIN (long-lasting insecticidal net) distribution (not on an annual basis), (6) educational programming, and (7) maternal and child health visits and screenings. The intervention has been described in detail^4–7^. The control condition, as a part of the National Vector Control Strategy, is standard of care (SOC) whereby Accredited Social Health Activists (ASHAs) conduct door-to-door fever surveillance weekly. Fever cases are tested with RDTs and treated; severe cases are referred to hospitals^8^. IRS is conducted twice in a year during the transmission season in selected high risk areas having an annual parasite index (API) > 5, and LLINs are distributed in high risk areas having API > 2^9^. Thus key distinguishing features of MCs versus SOC include MSAT (everyone is offered malaria screening regardless of fever status in the MCs), educational programming, and maternal and child health visits and screenings.

Odisha state has seen a > 80% decline in malaria cases since 2017, attributed to the large-scale distribution of LLINs and the DAMaN program^10^. The aim of our project was to evaluate the effectiveness of the DAMaN MCs through a quasi-experimental cluster-assigned (i.e., non-randomized) stepped-wedge study with a pretest–posttest control group design.

## Results

We enrolled 2463 participants into three study arms: six immediate interventions (Arm A), six delayed interventions (Arm B), and three previous interventions (Arm C), and sampled them at baseline, with three follow-ups from August 2019 to December 2020. The primary outcome was PCR + *Plasmodium* infection prevalence. A time (i.e., visit) $\times$ study arm interaction revealed statistically significant lower odds of PCR + malaria in Arm A versus B at the third follow-up. Our results suggest that the DAMaN program’s malaria camps were associated with lower malaria incidence relative to standard-of-care.

### Study population and study design

**Figure 1:**
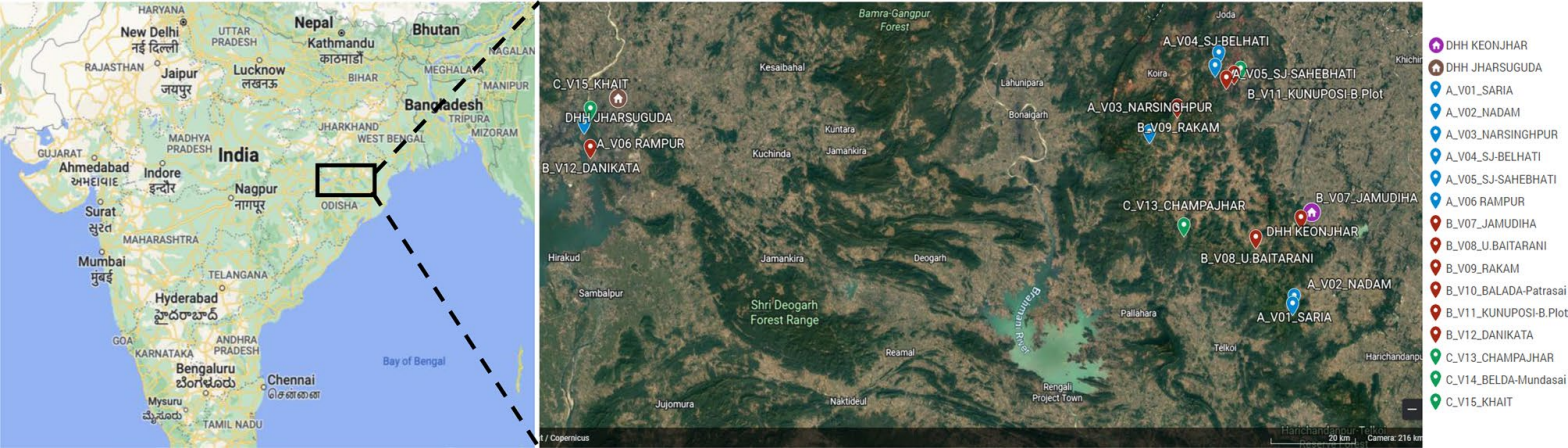
Location of 15 hard-to-reach villages in Odisha, India. The two DHH (district headquarter hospitals) are indicated, and each village is named with its study arm letter (A, B, C) and village number (V1–15) provided. Maps created with Google©2023, INEGI.

**Figure 2:**
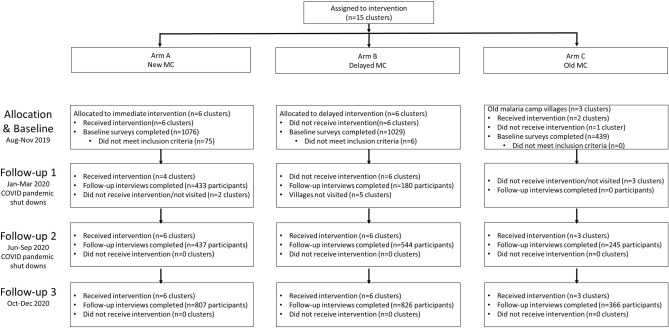
Cluster-assigned quasi-experimental study of malaria camps as part of the DAMaN malaria elimination program in Odisha, India. Flow chart providing a summary of the 15 clusters (villages) distributed between three arms and sampled at baseline and three follow-ups. A timeline of the activities is also given, and whether COVID-19 shutdowns are known to have occurred.

Fifteen villages were selected in the northern districts of Keonjhar and Jharsuguda in Odisha state, India, in consultation with the Odisha Malaria Control Program (Fig. 1). The villages were distributed between three study arms: six villages in Arm A ‘new-MC’ (communities receiving MCs for the first time in year one), six villages in Arm B ‘delayed-MC’ (communities undergoing routine malaria control in year one and receiving MCs for the first time in year two), and three villages in Arm C ‘old-MC’ villages, where MCs had already been implemented prior to the study period. A flow chart of the study is shown in Fig. 2.

We enrolled 2463 participants in the study and Table 1 presents their baseline demographic characteristics. A total of 14.1% of the participants were aged 5 years or younger, 29.4% were aged 6 to 17, 23.6% were 18–34, and 32.8% were aged 35 or older, with females comprising 56.1% of the participants. The participants generally had low educational attainment: almost half (49.3%) had no schooling/less than primary school whereas 5.7% had higher secondary school or more. With respect to occupation, 28.8% were housewives, 34.9% were students, 5.6% were farmers/agricultural laborers, 18.6% were employed in another trade, 9.6% were children that were not in school, and 2.6% did not have an occupation.

**Table 1:** Demographic characteristics and *Plasmodium* outcomes at baseline and the third follow-up visit.

Variable	Total sample N = 2463 n (%)	Arm A New MC n = 1001 n (%)	Arm B Delayed MC n = 1023 n (%)	Arm C Old MC n = 439 n (%)	p-value^1^
Age categories					< 0.001
≤ 5	348 (14.1)	126 (12.6)	147 (14.4)	75 (17.1)	
6–17	725 (29.4)	254 (25.4)	309 (30.2)	162 (36.9)	
18–34	581 (23.6)	251 (25.1)	235 (23.0)	95 (21.6)	
≥ 35	809 (32.8)	370 (37.0)	332 (32.5)	107 (24.4)	
Sex					0.063
Male	1079 (43.9)	466 (46.7)	427 (41.7)	186 (42.4)	
Female	1381 (56.1)	532 (53.3)	596 (58.3)	253 (57.6)	
Missing	3	3	0	0	
Educational attainment					< 0.001
No schooling/ less than primary	1214 (49.3)	486 (48.6)	510 (49.9)	218 (49.7)	
Primary	561 (22.8)	228 (22.8)	221 (21.6)	112 (25.5)	
Middle, secondary, or matric	547 (22.2)	204 (20.4)	254 (24.8)	89 (20.3)	
Higher secondary or more	140 (5.7)	82 (8.2)	38 (3.7)	20 (4.6)	
Missing	1	1	0	0	
Occupation					< 0.001
Farmer or agricultural laborer	138 (5.6)	76 (7.6)	37 (3.6)	25 (5.7)	
Other employment or trade	457 (18.6)	243 (24.3)	170 (16.6)	44 (10.0)	
Housewife	709 (28.8)	266 (26.6)	326 (31.9)	117 (26.7)	
Student	858 (34.9)	289 (28.9)	391 (38.2)	178 (40.5)	
Child, not in school	236 (9.6)	93 (9.3)	82 (8.0)	61 (13.9)	
None	63 (2.6)	32 (3.2)	17 (1.7)	14 (3.2)	
Missing	2	2	0	0	
Follow-up rate for third follow-up	1999 (81.2)	807 (80.6)	826 (80.7)	366 (83.4)	0.425
**Baseline**					
Any *Plasmodium* infection by RDT					0.014
No	2406 (98.1)	964 (97.3)	1005 (98.2)	437 (99.5)	
Yes	47 (1.9)	27 (2.7)	18 (1.8)	2 (0.5)	
Missing	10	10	0	0	
**Third follow-up visit**					
Any *Plasmodium* infection by RDT					
No	1983 (99.3)	804 (99.6)	813 (98.7)	366 (100.0)	0.017^3^
Yes	14 (0.7)	3 (0.4)	11 (1.3)	0 (0.0)	
Missing	2	0	2	0	
p-value^2^	< 0.001	< 0.001^3^	0.466	0.503^3^	

MC = malaria camp; RDT = rapid diagnostic test.

^1^χ^2^ p-value for comparisons across arms excluding missing, unless otherwise noted.

^2^χ^2^ p-value for comparisons between baseline and follow-up 3 excluding missing, unless otherwise noted.

^3^Fisher’s exact test.

Across the study arms there were statistically significant differences in age, educational attainment, and occupation, but not sex. As evidenced by the age category distribution and the mean age for each arm, Arm A was older (mean age [M_age_] = 27.2, standard deviation [SD] = 19.0) and Arm C was younger (M_age_ = 22.2, SD = 18.8) than Arm B (M_age_ = 25.7, SD = 19.6, p_ANOVA_ < 0.001). A higher proportion of participants in Arm A had higher secondary education or higher while a higher proportion in Arm B had middle, secondary, or matric and in Arm C a high proportion had primary education. There were a higher proportion of farmers/agricultural laborers and people with other employment/trades in Arm A, a higher proportion of housewives in Arm B, and higher proportions of students and children not in school in Arm C. Given the differences across arms, adjusted odds ratios (aORs) control for demographics.

### Intent-to-treat analysis for primary and secondary malaria outcomes

The MC intervention was revised from our initial protocol paper^7^ with two major and consistent differences. First, all MCs conducted MSAT rather than FSAT in all rounds of the intervention. Second, LLINs were not distributed in our study villages because their 3-yearly replenishment distribution scheduled for 2020 was delayed due to the Covid-19 pandemic.

**Figure 3:**
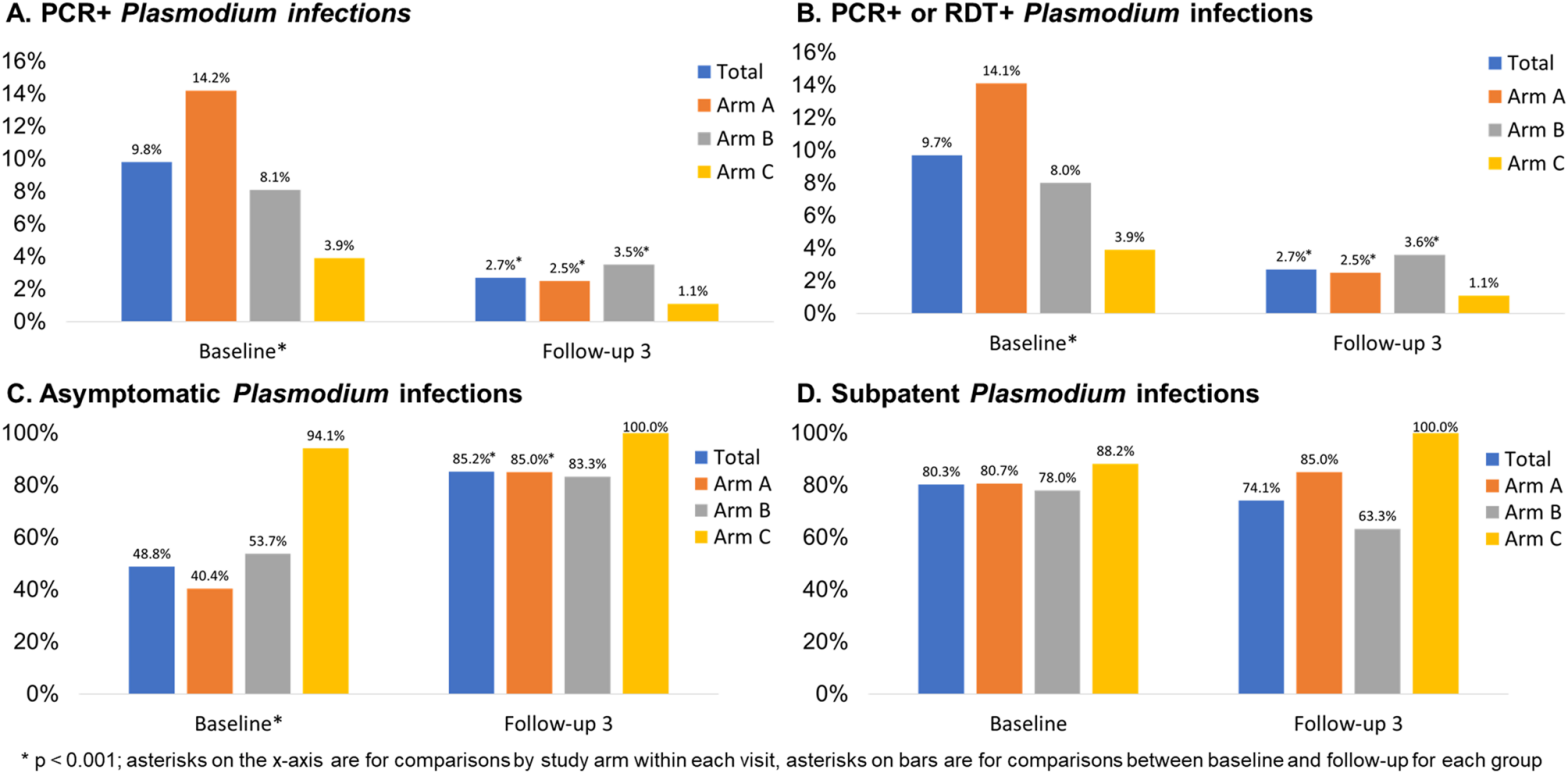
*Plasmodium* infection outcomes at baseline and follow-up 3 by study arm, Odisha, India. Four panels of data showing: (A) PCR + and (B) PCR + or RDT + *Plasmodium* infection prevalence at baseline (n = 2463) and follow-up 3 (n = 1999); (C) asymptomatic *Plasmodium* infection prevalence at baseline (among n = 240 total infections) and follow-up 3 (among n = 54 total infections); (D) subpatent *Plasmodium* infection prevalence at baseline (among n = 240 total infections) and follow-up 3 (among n = 54 total infections).

Table 1 presents the malaria outcomes at baseline and the third follow-up (FU3) visit; associations are considered statistically significant if p < 0.013, based on a Bonferroni correction. At baseline, 9.8% of the sample had PCR + *Plasmodium* infection, with statistically significant differences across study arms. Arm A had the highest prevalence of *Plasmodium* infection (14.2%), followed by Arm B (8.1%) and Arm C (3.9%, p < 0.001) (Fig. 3, panel A). Prevalence of RDT + *Plasmodium* infection was 1.9% and there were no significant differences across arms; Arm A again had the highest prevalence of *Plasmodium* infection (2.7%), followed by Arm B (1.8%) and Arm C (0.5%, p = 0.014). When malaria was defined by any positive PCR or RDT test, prevalence overall was 9.7% at baseline. With respect to species, 236 (98.3%) were *P. falciparum* cases and 9 (3.8%) were *P. vivax*; 5 (2.1%) were co-infected with *P. falciparum* and *P. vivax* [data not shown]. Approximately 48.8% of *Plasmodium* infections were asymptomatic (defined as RDT + or PCR + and no fever) at baseline with Arm C having the highest proportion (94.1%) of asymptomatic cases as compared to Arms A and B (Fig. 3, panel C: 40.4 and 53.7%, respectively; p < 0.001). A substantial majority (80.3%) of *Plasmodium* cases were subpatent (i.e., RDT- and PCR +), with no significant differences across arms (Fig. 3, panel D).

At FU3, 2.7% of the sample had PCR + *Plasmodium* infection, with no significant differences across arms (Fig. 3, panel A). Prevalence of RDT + *Plasmodium* infection was 0.7% overall at follow-up, with 0.4% prevalence in Arm A, 1.3% prevalence in Arm B, and no observed cases in Arm C Table 1**,** p = 0.017). When malaria was defined by any positive PCR or RDT test, prevalence overall was 2.7% at FU3 (Fig. 3, panel B). When we compared malaria outcomes from baseline to follow-up for the entire sample as well as each arm, we found that there was a statistically significant reduction in both RDT + and PCR + *Plasmodium* infection in the sample overall and for Arm A. We observed a statistically significant reduction in PCR + but not RDT + *Plasmodium* infection in Arm B and no significant differences in Arm C. With respect to species, 53 (98.1%) were *P. falciparum* cases and 1 (1.9%) was *P. vivax*; none were co-infected with *P. falciparum* and *P. vivax* at FU3. Approximately 85.2% of *Plasmodium* infections were asymptomatic at FU3 with no significant differences across arms but statistically significant increases in the proportion of cases that were asymptomatic from baseline to FU3 for the entire sample (Fig. 3, panel C, p < 0.001), Arm A (p =  < 0.001), and Arm B (p = 0.004) but not Arm C. A substantial majority (74.1%) of *Plasmodium* cases were subpatent at FU3, with no significant change from baseline to FU3 (Fig. 3, panel D).

The overall follow-up rate for FU3 was 81.2% with a range of 80.6 to 83.4% across arms, with no significant differences across arms (Table 1). The sociodemographic and malaria outcome correlates of participants lost-to-follow-up (LTFU) is shown in Supplemental Table 1. There were no significant differences in LTFU by study arm or sex. Younger people were more likely to be LTFU as were those with lower educational attainment, students, and children not in school. Those with malaria, regardless of detection method, were statistically significantly more likely to be LTFU but there were no significant differences by asymptomatic or subpatent cases.

Table 2 presents the crude and adjusted multilevel mixed effects logistic regression models for the primary and secondary malaria outcomes. The adjusted models controlled for baseline *Plasmodium* infection, age, and gender given baseline differences in *Plasmodium* infection prevalence and demographics across study arms. Education and occupation were not included in the model, although they were statistically significant in the bivariate analyses, due to multicollinearity. The time (i.e., baseline vs. FU3) x study arm interaction term shows that there were statistically significant lower odds of PCR + *Plasmodium* infection in Arm A (AOR = 0.36, 95% CI = 0.17, 0.74) but not Arm C as compared to Arm B at follow-up. The adjusted model for PCR + or RDT + *Plasmodium* infection showed similar results. For RDT + *Plasmodium* infection, odds ratios could not be calculated due to small numbers of RDT + *Plasmodium* infections at FU3. Village-specific *Plasmodium* infection detected by PCR at baseline and FU3 are provided in Supplemental Table 2.

**Table 2:** Multilevel mixed-effects logistic regression models estimating the association between malaria camps and RDT + and/or PCR + *Plasmodium* infections.

Outcome	*Plasmodium* infection by PCR only		*Plasmodium* infection by RDT or PCR	
	Crude OR (95% CI)	Adjusted OR (95% CI)^1^	Crude OR (95% CI)	Adjusted OR (95% CI)^1^
Plasmodium infection at follow-up 3 by study arm				
Arm B Delayed MC	1.0	1.0	1.0	1.0
Arm A New MC	0.34 (0.16, 0.71)	0.36 (0.17, 0.74)	0.33 (0.16, 0.69)	0.35 (0.17, 0.72)
Arm C Old MC	0.63 (0.17, 2.27)	0.64 (0.18, 2.30)	0.62 (0.17, 2.22)	0.63 (0.18, 2.25)
Model fit statistics				
Log likelihood	−914.96	−902.38	−922.53	−910.12
Model p-value	< 0.001	< 0.001	< 0.001	< 0.001
Akaike's Information Criteria (AIC)	1845.92	1828.77	1861.07	1844.24
Bayesian Information Criteria (BIC)	1897.08	1905.48	1912.30	1921.05

RDT = rapid diagnostic test; PCR = polymerase chain reaction; OR = odds ratio; CI = confidence interval; MC = malaria camp.

^1^Adjusted for baseline *Plasmodium* infection, age, and gender.

### Intent-to-treat analysis for secondary outcomes

The anthropometric and clinical outcomes at baseline and the third follow-up visit are shown in Table 3; associations are considered statistically significant if p < 0.013. At baseline, 2.4% were severely underweight, with no differences across study arms. At follow-up, the prevalence of severe underweight decreased to 1.5%; there were still no differences across arms in underweight at follow-up. Few (2.2%) were severely anemic, with significantly more anemia in Arm A (5.1%) as compared to Arms B and C (0.1 and 0.7%, respectively; p < 0.001). At follow-up there were no significant differences in anemia across arms, but there were statistically significant decreases from baseline to follow-up for the total sample and Arm A.

**Table 3:** Anthropometric outcomes at baseline and the third follow-up visit.

Variable	Total sample N = 2463 n (%)	Arm A New MC n = 1001 n (%)	Arm B Delayed MC n = 1023 n (%)	Arm C Old MC n = 439 n (%)	p-value^1^
**Baseline**					
Severe underweight					0.093
No	2378 (97.6)	952 (97.2)	1002 (98.3)	424 (96.6)	
Yes	59 (2.4)	27 (2.8)	17 (1.7)	15 (3.4)	
Missing	26	22	4	0	
Severe anemia					< 0.001^2^
No	2388 (97.8)	940 (94.9)	1014 (99.9)	434 (99.3)	
Yes	54 (2.2)	50 (5.1)	1 (0.1)	3 (0.7)	
Missing	21	11	8	2	
Fever					< 0.001
No	1861 (75.8)	851 (85.4)	632 (62.0)	378 (86.1)	
Yes	595 (24.2)	146 (14.6)	388 (38.0)	61 (13.9)	
Missing	7	4	3	0	
MUAC malnutrition^3^					< 0.001
No	1982 (80.7)	813 (81.8)	846 (82.8)	323 (73.6)	
Yes	473 (19.3)	181 (18.2)	176 (17.2)	116 (26.4)	
Missing	8	7	1	0	
**Third follow-up visit**	1999 (81.2)	807 (80.6)	826 (80.7)	366 (83.4)	0.425
Severe underweight					0.526^2^
No	1960 (98.5)	790 (98.3)	811 (98.4)	359 (99.2)	
Yes	30 (1.5)	14 (1.7)	13 (1.6)	3 (0.8)	
Missing	9	3	2	4	
p-value^4^	0.031	0.154	0.878	0.016^2^	
Severe anemia					0.592^2^
No	1991 (99.8)	805 (99.9)	821 (99.9)	365 (99.7)	
Yes	3 (0.2)	1 (0.1)	1 (0.1)	1 (0.3)	
Missing	5	1	4	0	
p-value^4^	< 0.001^2^	< 0.001^2^	1.000^2^	0.630^2^	
Fever					0.225^2^
No	1982 (99.3)	799 (99.1)	818 (99.3)	365 (100.0)	
Yes	13 (0.7)	7 (0.9)	6 (0.7)	0 (0.0)	
Missing	4	1	2	1	
p-value^4^	< 0.001	< 0.001	< 0.001	< 0.001^2^	
MUAC malnutrition^3^					0.004
No	1658 (83.1)	693 (85.9)	681 (82.7)	284 (78.0)	
Yes	336 (16.9)	114 (14.1)	142 (17.3)	80 (22.0)	
Missing	5	0	3	2	
p-value^4^	0.038	0.020	0.985	0.144	

^1^χ^2^ p-value for comparisons across arms excluding missing, unless otherwise noted.

^2^Fisher’s exact test.

^3^ < 23 cm for adults and adolescents aged 15 and older, < 11.5 cm for children 6 to 60 months, < 12.9 cm for 5–9 year olds, < 16 cm for 10–14 year olds.

^4^p-value for comparing baseline to follow-up 3 excluding missing, unless otherwise noted.

MC = malaria camp; MUAC = mid-upper arm circumference.

At baseline, prevalence of fever was 24.2%. Arm B had the highest prevalence (38.0%) as compared to Arm A (14.6%) and Arm C (13.9%, p < 0.001). There were no significant differences in fever across arms at follow-up, although there were statistically significant reductions from baseline to follow-up overall and for each arm (p < 0.001). Prevalence of malnutrition measured by mid-upper arm circumference (MUAC) was 19.3% at baseline, with higher prevalence in Arm C (26.4%) as compared to Arms A (18.2%) and B (17.2%, p < 0.001). At follow-up there were statistically significant differences in malnutrition across arms but no significant differences from baseline to follow-up. Crude and adjusted multilevel mixed effects logistic regression models were not estimated for these secondary outcomes due to small numbers (i.e., severe underweight, severe anemia, fever) and/or few significant differences in the bivariate analyses comparing baseline to follow-up (i.e., severe anemia and MUAC malnutrition).

### Malaria exposure and serology in a subset of study participants

We assayed 285 baseline samples (238 PCR + and 47 PCR-) for anti-*Plasmodium* antibodies to 13 *P. falciparum*-specific antigens and four *P. vivax*-specific antigens using the Luminex MAGPIX platform and xPONENT software to estimate mean fluorescence intensity (MFI). Data were missing for six markers (n = 174 missing for PvMSP10; n = 20 missing for PfEBA140 R3-5, PfETRAMP5_ag1, PfMSP2CH150, PfRh2_2030, and PvMSP8). There were no significant differences by gender for *P. falciparum* and *P. vivax* antibodies, but the proportion positive increased as age increased (data not shown).

Table 4 summarizes the antibody results by study arm and infection status. There were no significant differences between Arms A and B in terms of *P. falciparum* antibodies at baseline; a higher proportion of Arm B participants had *P. vivax* antibodies at baseline compared to Arm A participants. There were statistically significant differences in antibodies across all three arms, with a lower proportion of Arm C participants having *Plasmodium* antibodies. With respect to baseline infection status, a higher proportion of symptomatic participants had *P. falciparum* antibodies and *P. vivax* antibodies as compared to uninfected and asymptomatic participants.

**Table 4:** Baseline *Plasmodium* antibodies among 238 PCR + and 47 PCR- participants in the Phase 1 study, by study arm and infection status.

	Any Pf antibodies (n = 177)		Any Pv antibodies (n = 77)		Any Pf or Pv antibodies (n = 178)	
	n (%)	p	n (%)	p	n (%)	p
Study arm						
Arm A (New MC)	98 (60.9)	0.010*	40 (24.8)	0.002*	99 (61.5)	0.011*
Arm B (Delayed MC)	69 (70.4)	0.120**	36 (36.7)	0.042**	69 (70.4)	0.145**
Arm C (Old MC)	10 (38.5)		1 (3.8)		10 (38.5)	
Infection status		< 0.001		0.003		< 0.001
Uninfected	26 (55.3)		13 (27.7)		26 (55.3)	
Symptomatic	91 (77.8)		43 (36.8)		91 (77.8)	
Asymptomatic	60 (49.6)		21 (17.4)		61 (50.4)	

* Arm A vs. B vs. C ** Arm A vs. B PCR = polymerase chain reaction; Pf = *Plasmodium falciparum;* Pv = *Plasmodium vivax*; MC = malaria camp.

### Village-level costs of MCs

We conducted an analysis of the implementation costs of MCs in the study villages from the service provider perspective. The results of the cost analysis are presented in Table 5. In the study villages, the cost per person participated ranged between US$3–8, the cost per tested between US$4–9, and the cost per treated between US$82–1614 per camp round.

**Table 5:** Village-level costs of the DAMAN intervention in study villages in Odisha State, India (all costs are in 2020 US dollars; *per person).

Village	V01	V02	V03	V04/V05	V06	V07	V08	V09	V10	V11	V12	V13	V14	V15
Population participated	407	208	535	700	507	254	313	547	146	250	510	381	380	266
Population tested	387	187	511	479	444	216	293	410	137	238	448	362	235	247
Population treated	1	2	22	2	0	0	0	22	0	0	0	2	0	0
Personnel costs (%)	1001 (62)	1001 (76)	1001 (55)	1001 (49)	1001 (57)	1001 (72)	1001 (68)	1001 (55)	1001 (82)	1001 (73)	1001 (57)	1001 (64)	1001 (64)	1001 (71)
Implementation costs (%)	613 (38)	313 (24)	806 (45)	1055 (51)	764 (43)	383 (28)	472 (32)	824 (45)	220 (18)	377 (27)	768 (43)	574 (36)	574 (36)	401 (29)
Total cost (%)	1614 (100)	1314 (100)	1807 (100)	2056 (100)	1765 (100)	1384 (100)	1473 (100)	1825 (100)	1221 (100)	1378 (100)	1769 (100)	1575 (100)	1575 (100)	1402 (100)
Cost per person participated*	4	6	3	3	3	5	5	3	8	6	3	4	4	5
Cost per person tested*	4	7	4	4	4	6	5	4	9	6	4	4	7	6
Cost per person treated*	1614	657	82	1028	.	.	.	83	.	.	.	787	.	.

*2020 US dollars (US$).

## Discussion

In this quasi-experimental study conducted in rural villages in Odisha, intent-to-treat analyses suggest that the DAMaN program’s malaria camps were associated with lower malaria incidence relative to standard-of-care. This is suggested by the reductions in *Plasmodium* infection prevalence at baseline versus FU3. We note that all villages received MCs at some point, with Arm A receiving MCs four times, Arm B receiving it twice, and Arm C receiving it prior to the study’s inception and three times during the study. Intervention activities were impacted by the COVID-19 pandemic-related shutdowns and, as a result, 9 villages (2 of 6 in Arm A, 6 of 6 in Arm B, and 2 of 3 in Arm C) did not receive any malaria camp activities during the first follow-up. There were no serious adverse events.

Our findings are in contrast to a recent meta-analysis that noted a marginal and non-significant pooled effect size for MSAT interventions on *Plasmodium* infection prevalence and incidence^11^. One major limitation of previous evaluations of MSAT is low sensitivity of the diagnostic methods (i.e., RDTs and light microscopy)^11^. The Odisha DAMaN MCs use RDTs for MSAT while this evaluation used both an RDT and PCR, overcoming some previous evaluations’ limitations.

There were no significant differences in the subset of Arm A and B participants tested for *Plasmodium* antibodies, suggesting that Arms A and B had similar malaria exposure prior to the intervention. There were statistically significant differences in *Plasmodium* antibodies across all 3 arms, with a lower proportion of Arm C participants having antibodies; this suggests lower transmission of *Plasmodium* in these villages and/or impact of the previous exposure to the MC intervention.

At baseline, more symptomatic (i.e., febrile) participants had *P. falciparum* and *P. vivax* antibodies as compared to uninfected and asymptomatic participants. These results suggest possible cross-reactivity of *P. falciparum* and *P. vivax* antibodies and antigens in the assay as well as potential previous exposure to *P. vivax*. The lower proportions of participants with *P. vivax* antibodies relative to *P. falciparum* antibodies is consistent with RDT and PCR data demonstrating lower prevalence of *P. vivax* in this study sample, as well as data from the National Center for Vector Borne Diseases Control showing that 91.4% of cases in 2021 were *P. falciparum* infections^3^.

The Malaria Control Program implemented MSAT across all malaria camp rounds rather than FSAT in the subsequent rounds. This likely contributed to the impact of the intervention, as 49 of the 69 RDT + cases (71.0%) detected in the study were afebrile [data not shown]. In other words, they treated more *Plasmodium* infections than they otherwise would have if they had only tested and treated febrile cases each time.

The cost per person for the MCs ranged between US$3–8, the cost per tested person between US$4–9, and the cost per treated person between US$82–1,614 per MC round. According to our systematic literature review on the effectiveness and cost-effectiveness of intermittent mass MSAT interventions for malaria^11^, these results are comparable to the costs reported in the published literature, ranging between US$3.5–14.3 per person tested per round, and corresponds roughly to 20–35% of the per-capita domestic general health expenditure in India (US$19.6 in 2018)^12^.

We note several important study limitations. First, as discussed in our study protocol^7^, the villages were not randomized to study arms, which may result in potential selection bias. Second, COVID-19 pandemic shutdowns resulted in disruption in delivery of the MCs at FU1 which meant that several villages may not have received all or any of the activities in that MC round. Third, while the follow-up was over 80%, people with *Plasmodium* infection at baseline were less likely to be followed-up but there were no significant differences in follow-up among asymptomatic or subpatent cases.

Collectively, our quasi-experimental cluster-assigned stepped-wedge study results suggest that the DAMaN malaria camp intervention is associated with reductions in malaria in rural villages and thus is a promising, financially feasible approach for malaria control in rural settings. However, given the quasi-experimental design and COVID-related study challenges, we cannot infer causality. This is timely considering that Odisha State government extended the DAMaN initiative in October 2022 for five more years in 21 districts in a bid to achieve malaria elimination in Odisha by 2030^13^.

## Methods

### Ethics statement

Ethical approval for the trial was obtained from the Odisha State Research and Ethics Committee (Odisha, India, dated 24 June 2019), institutional ethics committee at Community Welfare Society Hospital (Rourkela, Odisha, India) and the institutional review board at New York University (New York, NY, USA). All research was performed in accordance with relevant guidelines/regulations and the Declaration of Helsinki.

### Study design and enrollment procedures

The quasi-experimental cluster-assigned stepped-wedge study design, power analysis, and recruitment/enrollment procedures are described in a protocol paper^7^ and the study was registered at ClinicalTrials.gov (NCT03963869; first posted 28 May 2019). Briefly, 12 intervention villages were selected in two districts of Odisha state, Keonjhar and Jharsuguda, and distributed in equal numbers between two study arms: Arm A ‘new-MC’ (communities receiving MCs for the first time in year one) and Arm B ‘delayed-MC’ (communities undergoing routine malaria control in year one and receiving MCs for the first time in year two); control Arm C contained three ‘old-MC’ villages, where MCs had already been implemented prior to the study period. A cohort was planned with target enrollment of 2,700 individuals across all 15 villages (i.e., 1100 in Arms A and B; 500 in Arm C).

Study participants in each village were recruited and enrolled at baseline before the initial administration of the intervention by the Government of Odisha DAMaN team and subsequently surveyed prior to each round of their screen and treat program. RDT diagnosis and treatment of the study participants followed the Government of Odisha Department of Health and Family Welfare Standard Treatment Guidelines (https://health.odisha.gov.in/sites/default/files/2020-02/STG-2018.pdf). Study participants in the three villages not receiving the intervention were recruited and enrolled in parallel temporally. Residents of the study villages aged 1–69 were eligible for the study. Written informed consent was obtained in the local language from all adult study participants aged 18–69 years with apparent full comprehension of the study procedures. Assent was obtained in addition to parental or legal guardian informed consent for participants aged 7–17 years. Parental informed consent was obtained for children aged 1–6 years. During each survey round, all subjects completed a health questionnaire that captured demographic, malaria knowledge and prevention, and *Plasmodium* infection and treatment history data and provided a blood sample for *Plasmodium* parasite detection.

### Intervention adaptations due to COVID-19 pandemic

Arm A (new MC) and C (old MC) villages were to receive two cycles of MCs, each with two rounds of MSAT. The first MC cycle was from August to November 2019 (Round 1) and January to March 2020 (Round 2). We note that the Round 2 MSAT was interrupted by COVID-19 pandemic shutdowns. The second MC cycle was from June to September 2020 (Round 1) and October to December 2020 (Round 2). We note that Round 1 MSAT was also interrupted by COVID-19 pandemic shutdowns. Arm B (delayed MC) villages were to receive SOC in the first cycle (August 2019–March 2020) and one MC cycle with two rounds of MSAT from June to September 2020 (Round 1) and October to December 2020 (Round 2). Again, Round 1 was interrupted by the pandemic; see Supplemental Fig. 1 for details.

### Primary outcomes

The primary outcome measures were any *Plasmodium* as detected by PCR and *Plasmodium* species (i.e., *P. falciparum* and/or *Plasmodium vivax*) as detected by PCR. Secondary malaria outcome measures included *Plasmodium* infection as detected by RDT and *Plasmodium*-specific serology. *Plasmodium* infection by RDT is defined as RDT negative (RDT−) or RDT positive (RDT+). We further classified infections as ‘asymptomatic’, defined as PCR+ and/or RDT+ for any *Plasmodium* species with absence of documented fever or self-reported fever in the last 48 h, and/or ‘subpatent’, defined as RDT− and PCR+. *Plasmodium*-specific serology was analyzed as a continuous antibody titer variable as well as a categorical (seropositive vs. seronegative) variable.

### Blood sample collection and processing

Blood samples for blood smears, blood spots (Whatman FTA cards), a small volume microvette, and micro volumes required for point of care hemoglobin and RDTs (FalciVax), were collected from consenting study participants by sterile lancet finger prick as previously described^7^. Vacutainers (5 ml; BD Vacutainer glass blood collection tubes with acid citrate dextrose) of venous blood were requested from study participants found to be positive by RDT. Microvette and vacutainer samples were refrigerated until separated into plasma and infected red blood cell (iRBC) components by centrifugation. Plasma was stored at −80 °C until assayed for *Plasmodium* immunoglobulin G (IgG), and total DNA was extracted from the iRBC pellet using QIamp DNA mini kit (QIAGEN) with a final elution volume of 50 μl.

### Molecular detection of Plasmodium parasites by species-specific PCR

DNA samples were tested for *Plasmodium* species by a PCR assay targeting multi-copy loci Pfr364 and Pvr47^14^ as previously described^7^. Five μl of DNA was used in a total reaction volume of 30 μl, and amplification products were visually confirmed by ethidium bromide-stained gel electrophoresis using a Gel Doc EZ documentation system (BioRad Laboratories, Inc.).

### Detection of anti-Plasmodium IgG by Luminex MAGPIX cytometric bead array

Plasma isolated from a subset of microvette samples of finger prick blood was assayed for 13 anti-*P. falciparum* and four anti-*P. vivax* antibodies as previously described.^7^ The panel included antigens whose corresponding antibodies have been suggested as indicators of protection from clinical disease^15,16^, long-term or cumulative exposure^17–20^, and recent infection^17–20^. Briefly, n = 300 plasma samples were diluted 1/200 and assayed in duplicate according to standard procedures^21,22^. xPONENT software was used for data acquisition (Luminex Corp., Austin, TX). Specifically, the net Mean Fluorescence Intensity (MFI; net MFI_Ag_ = raw MFI_Ag_ − background MFI_Ag_ where background MFI_Ag_ is the mean MFI of a given antigen in the blank wells) was calculated for each antigen in each sample. The presence of antibody to each antigen (e.g., seropositivity) was defined as the mean net MFI_naive pool_ plus three standard deviations. Composite variables for any *P. falciparum*, any *P. vivax*, and any *P. falciparum* or *P. vivax* antibodies were created.

### Secondary anthropometric and clinical outcomes

We report on several anthropometric measurements, including severe underweight, severe anemia, fever, and malnutrition as measured by mid-upper arm circumference (MUAC). For adults aged 18 and older, severe underweight was defined as a BMI < 16. For children, severe underweight was defined as three standard deviations below the median based on the WHO growth charts used for children < 5 years and the revised Indian Academy of Pediatrics (IAP) growth charts for Indian children and adolescents aged 5–17^23^. Severe anemia was defined as hemoglobin < 5 g/dl for persons aged < 12 and 7 g/dl among those aged 12 and older^24^. Fever was defined as a temperature of 99.5°F or higher. MUAC malnutrition was defined as < 23 cm for adults and adolescents aged 15 and older^25,26^, < 16 cm for 10–14 year olds^27^, < 12.9 cm for 5–9 year olds^27^, and < 11.5 cm for children 6 to 60 months^28^.

### Costing study design, data collection, and analyses

Using an activity-based costing method, we conducted an analysis of the implementation costs of malaria camps (MCs) in the study villages from the service provider perspective^29^. Interviews were conducted with the malaria camp supervisors who led the MCs in the study villages, and programmatic documentation from the Odisha State malaria control program were reviewed to identify the activities related to the planning and implementation of DAMaN at the village-level. These included: (1) Training activities (training of public health supervisors and workers as well as village volunteers, including the village specific ASHAs; (2) Micro-plan development for MCs; (3) Planning and sensitization activities (identification of village volunteers, selection of camp venue and time); (4) Community sensitization activities; (5) Community mobilization activities; (6) Health activities (malaria testing and treatment, child health, maternal health); (7) Monitoring and supervision activities; and (8) Follow-up activities. Resource use was linked to specific activities associated with an intervention. During interviews, the MC supervisors identified the time dedicated by MC staff to different activities for each camp round. Time costs of MC staff were calculated based on average annual gross wage rates, apportioned according to time devoted to DAMaN activities per camp round, and summed across all MC staff. Unit costs of rapid diagnostic kits and antimalarial drugs were extracted from the DAMaN Operational Guidelines^30^. The costs of these commodities were calculated by multiplying the quantities used during a camp round by their unit costs based on participation in each camp round in each village. Personnel and implementation costs per round were summed and divided by the population that participated in the MC, the population tested for malaria, and the population treated for malaria, in each study village to calculate cost per person participated, cost per person tested, and cost per person treated per camp round. All costs were collected in local currency and converted to and presented in 2020 US dollars (US$), using the average exchange rate for 2020 (1 US$ = 74.27 Indian Rupees)^31^.

### Statistical analyses

The primary outcome was *Plasmodium* presence and species as detected by PCR at the third follow-up visit. Secondary outcomes included *Plasmodium* presence and species as detected by RDT and anthropometric and clinical outcomes including underweight, anemia, fever, and malnutrition as measured by mid-upper arm circumference (MUAC) at the third follow-up visit. Missing data was minimal among the analytic sample and ranged from 0 to 1.5% depending on the variable.

Bivariate analyses compared outcomes and key covariates across study arms with Pearson’s χ^2^ or Fishers exact tests (if cell sizes were < 5) at baseline and outcomes at follow-up 3. We use a Bonferroni adjusted α of 0.013 to account for multiple comparisons in the bivariate analyses comparing baseline to follow-up outcomes. Follow-up rates were calculated as the proportion of baseline participants that were followed up at the third time point.

Multilevel mixed-effects logistic regression was used to estimate the intervention effects, which enabled us to account for the repeated measure and nesting of individuals within the 15 villages. Mixed models are recommended when there are at least 10 clusters and fewer than 40 clusters^32,33^. The equation is:

$${y}_{ij}^{*}={\beta }_{0}+{{\beta }_{1}study\, arm}_{ij}+{{\beta }_{2}visit\,number}_{ij}+{{\beta }_{3}{study \,arm}_{ij}*visit \,number}_{ij}+{{\beta }_{4}age}_{ij}+{{\beta }_{5}sex}_{ij}+{{\beta }_{6}education}_{ij}{+ {\beta }_{7}occupation}_{ij}+{u}_{j}+{\epsilon }_{ij}$$

where ${y}_{ij}^{*}$ is the expected logit of plasmodium infection, $i$ is the individual, $j$ is the cluster (i.e., village), $\beta$ s are the fixed effects, ${u}_{j}$ is the random intercept for the villages, and ${\epsilon }_{ij}$ is the error term. Study arm, age, sex, education, and occupation are time-invariant covariates. Study arm is intent-to-treat, so even if a participant moved to another village, they remain assigned to the village where they were initially enrolled. Visit number in the only time-varying covariate. An interaction term (study arm x visit [baseline vs. FU3]) was used to estimate time effects for each arm and village was included as a random effect. We first estimated the crude associations for each outcome by study arm. We then adjusted these estimates for age, sex, education, and occupation. Odds ratios with 95% confidence intervals are reported. All analyses were conducted with Stata 17.0 (StataCorp, College Station, TX, USA) and were intent-to-treat. The study was registered at ClinicalTrials.gov (#NCT03963869).

### Supplementary Information

Supplementary Information.

## Data Availability

Epidemiology study data are available in the Clinical Epidemiology Database^34^ (https://clinepidb.org) under the ‘India ICEMR DAMaN Quasi-experimental Stepped-wedge’ study title.
