# Supplementary material for: The effectiveness of malaria camps as part of the malaria control program in Odisha, India
Source: Sci Rep. 2023 Dec 28;13:22998. doi: 10.1038/s41598-023-46220-x (PMC10754896; doi:10.1038/s41598-023-46220-x)
Supplement: Supplementary file 1 — Supplementary Information. [file 41598_2023_46220_MOESM1_ESM.pdf]

Supplemental files
The effectiveness of malaria camps as part of the malaria control program in Odisha, India

Danielle C. Ompad[1*], Timir K. Padhan.[2^], Anne Kessler[3^], Yesim Tozan[1], Abbey M. Jones[1], Anna Maria van Eijk[3], Steven A. Sullivan[3], Mohammed A. Haque[2], Madan Mohan Pradhan[4¶], Sanjib Mohanty[2¶], Jane M. Carlton[1,3¶] and Praveen K. Sahu[2¶*]

[1]School of Global Public Health, New York University, New York, NY, 10003, USA
[2]Department of Molecular Biology & Infectious Diseases, Community Welfare Society Hospital, Rourkela, Odisha, 769042, India
[3]Center for Genomics and Systems Biology, Department of Biology, New York University, New York, NY, 10003, USA
[4]Department of Health & Family Welfare, State Vector Borne Disease Control Programme, Bhubaneswar, Odisha, 751001, India
figure

[^]TKP and AK are joint second authors
¶PKS, JMC, SM, and MMP are joint last authors
*corresponding authors

Figure legends

Supplemental Figure 1. Timeline of the interventions and surveys (enrolments) undertaken in the study villages. Malaria Camp (MC) Interventions (Green) timeline and enrolment surveys (Orange) undertaken during Aug 2019 to Nov 2020 during the Covid-19 pandemic. BL: Baseline, FU1: Follow up 1, FU2: Follow up 2, FU3: Follow up 3. The timeline of village-wise enrolments is shown in the table: village-wise enrolments at baseline, FU1, FU2 and FU3 surveys. Enrolments were halted midway in FU1 (dark grey highlights) due to Covid 19 pandemic restrictions (designated ^) and impacted significantly in FU2 (light grey highlights) due to apprehension of being involved in the study by participants.

Supplemental Table 1. Demographic characteristics and *Plasmodium* outcomes for individuals retained in the study versus those lost-to-follow-up.

Variable	Lost-to-follow-up No n=1999 n (%)	Yes n=464 n (%)	p-value[1]
Study arm			
Arm A (New MC)	807 (40.4)	194 (41.8)	0.425
Arm B (Delayed MC)	826 (41.3)	197 (42.5)	
Arm C (Old MC)	366 (18.3)	73 (15.7)	
Age categories			
≤5	251 (12.6)	97 (20.9)	<0.001
6-17	569 (28.5)	156 (33.6)	
18-34	498 (24.9)	83 (17.9)	
>=35	681 (34.1)	128 (27.6)	
Sex			
Male	858 (42.9)	221 (47.6)	0.093[2]
Female	1139 (57.0)	242 (52.2)	
Missing	2 (0.1)	1 (0.2)	
Educational attainment			
No schooling/ less than primary	969 (48.5)	245 (52.8)	<0.001[2]
Primary	451 (22.6)	110 (23.7)	
Middle, secondary, or matric	447 (22.4)	100 (21.6)	
Higher secondary or more	131 (6.6)	9 (1.9)	
Missing	1 (0.1)	0 (0.0)	
Occupation			
Farmer or agricultural laborer	105 (5.3)	33 (7.1)	<0.001
Other employment or trade	384 (19.2)	73 (15.7)	
Housewife	614 (30.7)	95 (20.5)	
Student	684 (34.2)	174 (37.5)	
Child, not in school	162 (8.1)	74 (15.9)	
None	49 (2.5)	14 (3.0)	
Missing	1 (0.1)	1 (0.2)	
Any malaria by RDT			
No	1,981 (99.1)	425 (91.6)	<0.001[2]
Yes	11 (0.6)	36 (7.8)	
Missing	7 (0.4)	3 (0.6)	
Any *Plasmodium* infection by PCR			
No	1,924 (96.2)	264 (56.9)	<0.001
Yes	50 (2.5)	189 (40.7)	
Missing	25 (1.3)	11 (2.4)	
Any *Plasmodium* infection by RDT or PCR			
No	1,949 (97.5)	274 (59.1)	<0.001
Yes	50 (2.5)	190 (40.9)	
Asymptomatic *Plasmodium* infection[3]			
No	30 (60.0)	93 (48.9)	0.164
Yes	20 (40.0)	97 (51.1)	
Subpatent *Plasmodium* infection[4]			
No	11 (22.0)	36 (18.9)	0.754[2]
Yes	39 (78.0)	153 (80.5)	
Missing	0 (0.0)	1 (0.5)	

MC = malaria camp; RDT = rapid diagnostic test; PCR = polymerase chain reaction

[1] χ^2 p-value, unless otherwise noted
[2] Fishers exact test
[3] Asymptomatic *Plasmodium* infections are RDT+ or PCR+ and fever-
[4] Subpatent *Plasmodium* infections are RDT- and PCR+

Supplemental figure 1.

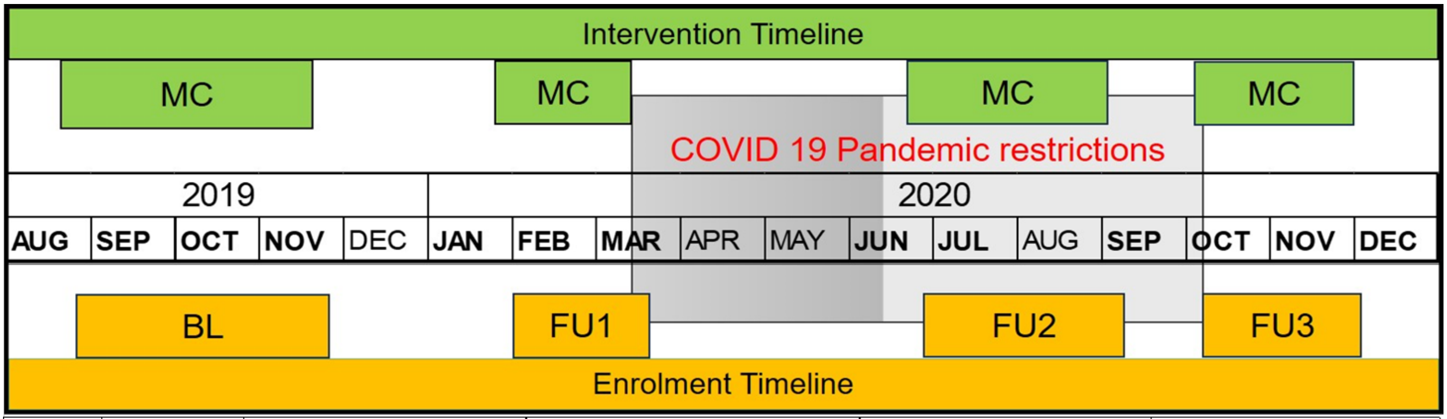

Arm	Village Code	Baseline	FU1	FU2	FU3
Arm A	V01	5-Sep-19	16-17 March 2020	26 & 27 June 2020	12 & 13 Nov 2020
	V02	22& 23 Aug 2019	Not done^	24 & 25 June 2020	20-Nov-20
	V03	26 & 27 Aug & 03 Sep 2019	Not done^	14 & 15 Jul 2020	04-06 Nov 2020
	V04	31-Aug-19	24 - 27 Feb 2020	03 & 04 Jul 2020	21 & 22 Nov 2020
	V05	1 & 3 Sep 2019	27-29 Feb & 01 March 2020	05 & 06 Jul 2020	23 & 24 Nov 2020
	V06	6-Sep-19	27-29 Jan 2020	03-05 Sep 2020	28 & 29 Oct 2020
Arm B	V07	03 & 04 Oct 2019	Not done^	30 June & 01 July 2020	16 & 17 Nov 2020
	V08	04 & 05 Oct 2019	Not done^	28 & 29 June 2020	18 & 19 Nov 2020
	V09	11 & 12 Oct 2019	Not done^	16 & 17 July 2020	07-09 Nov 2020
	V10	30 Sept & 01 Oct 2019	Not done^	09 & 10 July 2020	25 & 26 Nov 2020
	V11	13 & 14 Oct 2019	Not done^	07 & 08 July 2020	27 & 28 Nov 2020
	V12	19 & 22 Oct 2019	11-14 Mar 2020	31 Aug & 01& 02 Sep 2020	26 & 27 Oct & 02 Nov 2020
Arm C	V13	08 & 09 Nov 2019	Not done^	20 & 21 July 2020	10 & 11 Nov 2020
	V14	06 & 07 Nov 2019	Not done^	12 & 13 July 2020	29 & 30 Nov 2020
	V15	18 & 19 Nov 2019	Not done	06-08 Sep 2020	30 & 31 Oct 2020

Supplemental Table 2. Village-specific *Plasmodium* outcomes at baseline and follow-up.

Study Arm	Village Number	Prevalence (%) of Plasmodium infection detected by PCR	
		Baseline	Follow-up 3
Arm A (New MC)	1	20.51	3.12
	2	24.49	0.00
	3	20.82	7.41
	4	10.09	1.13
	5	8.27	0.00
Arm B (Delayed MC)	6	11.29	0.00
	7	22.58	0.00
	8	1.48	0.00
	9	17.01	18.71
	10	0.75	0.00
	11	1.55	0.00
Arm C (Previous MC)	12	6.33	0.00
	13	9.38	3.77
	14	0.00	0.00
	15	3.16	0.00
